# Primacy vs. recency effects: the dominant role of recent over past dental experience in dental anxiety

**DOI:** 10.1186/s12903-026-07941-3

**Published:** 2026-02-20

**Authors:** Bela Birkas, Botond Laszlo Kiss, Carlos M. Coelho, Pooya Pasandideh Rahvard, Andras N. Zsido

**Affiliations:** 1 Department of Behavioral Sciences, Medical School, University of Pécs, Szigeti str. 12., Pécs, Baranya H 7624 Hungary; 2 Institute of Psychology, University of Pécs, Pécs, Hungary; 3 Szentágothai Research Centre, University of Pécs, Pécs, Hungary; 4 Department of Psychology, University of the Azores, Ponta Delgada, Portugal; 5 University Research Center in Psychology (CUIP), Faculty of Human and Social Sciences, University of Algarve, Faro, Portugal; 6 Research Centre for Contemporary Challenges, University of Pécs, Pécs, Hungary

**Keywords:** Dental fear, Dental anxiety, Dental experience, Pain, Cognitive vulnerability, Sex differences, Dental phobia, Injection fear

## Abstract

**Background:**

Dental fear and anxiety (DFA) is a prevalent problem with multifactorial origins, including past traumatic experiences, cognitive vulnerabilities, and sociodemographic factors. Since most previous studies have focused on early or cumulative dental trauma and so, the relevance and significance of the most recent dental experience in shaping DFA is less explored. This study aimed to examine how recent, past, and childhood dental experiences, along with pain-related fear and demographic variables, predict DFA severity.

**Methods:**

A cross-sectional online survey was conducted with 802 Hungarian adults (mean age = 28.74; 78% women). Dental fear and anxiety (DFA) was assessed using three validated instruments: the Dental Anxiety Question (DAQ), the Short Dental Fear Question (SDFQ), and the Modified Dental Anxiety Scale (MDAS). Participants reported past dental experiences categorized by life stages into childhood, adulthood, and the past year. Hierarchical linear regression models were constructed for each DFA measure across the three temporal dimensions, adjusting for age, sex, and pain/distress during the last dental visit.

**Results:**

In all three timeframes, DFA was significantly associated with painful or traumatic dental experiences. However, pain and distress during the most recent dental visit was found to be the strongest predictor of DFA across all models (*p* < .001). Sex was a consistent predictor, with women reporting higher DFA levels (*p* < .001), and age showed a negative association in some models. Dental problems in childhood were also associated with DFA, but the predictive power decreased when recent experiences were also considered.

**Conclusions:**

DFA develops based on early or cumulative experiences but is also significantly influenced by the most recent dental visit, highlighting a dynamic interaction between trait-like and state-like anxiety processes. Our findings underscore the importance of ensuring positive, pain-free dental care to disrupt the reinforcement of DFA and prevent avoidance behavior.

## Introduction

Dental fear usually refers to the negative reactions towards perceived or real threats associated with dental treatment (e.g., pain), while dental anxiety is rather used to describe the negative emotional state dental patients may experience [1–3]. Since these are overlapping concepts in some respects and are repeatedly used interchangeably, their combination as a single term as dental fear and anxiety (DFA) is also widely used. This combined phrase is often used to summarize negative emotions related to dental procedures. DFA also presents a prevalent public health concern due to its significant implications for oral health, quality of life, and healthcare systems. Recent systematic reviews reported that dental fear and anxiety affect between 13% and 24% of the adult population, while 3–5% is affected by severe dental phobia [1–3]. More specifically, women consistently report higher levels of dental fear compared to men, potentially due to greater sensitivity to bodily threat cues and influences through socialization [4, 5]. Some results suggest that because the limited access to preventive dental care, lower socioeconomic status individuals encounter invasive or emergency dental procedures with higher likelihood, which predispose individuals to DFA [6].

In line with that, DFA was found to be associated with higher rates of avoidance or postponement of dental visits, presumably leading to poorer oral health outcomes, such as more caries, periodontal disease, and tooth loss [7, 8].

The multifactorial origins of DFA include traumatic dental experiences, classical conditioning, cognitive-emotional factors, and sociodemographic influences. According to classical conditioning, painful dental events can cause neutral stimuli—such as the sound of a drill—to become associated with pain [9, 10]. Repeated or intense negative experiences strengthen this link, leading the previously neutral stimulus to trigger anxiety on its own, even without actual discomfort, thereby reinforcing dental fear. DFA often emerges early in life and can persist into adulthood, impacting both oral health and avoidant behaviour. In a recent systematic review and meta-analysis, approximately 30% of children aged 2 to 6 years globally experience DFA, especially those without prior dental visit experience or with caries history [11]. This indicates a clear relationship between oral health status in childhood and the early development of dental fear and anxiety [11]. In line with this, children who attended dental visits irregularly, partially because of fear of pain, were found to receive more severe dental treatments, such as extractions and develop DFA more likely, while less severe and painful procedures were not associated with DFA [12]. Similarly, in adulthood, distressing dental experiences remain a dominant risk factor for pathological forms of DFA as they increase perception and fear of pain and negative beliefs and thoughts concerning subsequent dental treatment [13–15]. Poor self-rated oral health or avoidance behaviours, such as delaying visits until severe pain occurs, were also significantly associated with higher DFA, with surgical and restorative treatments being most feared [15, 16]. Pain associated with invasive dental procedures and fear of injection-related pain has been identified as a primary factor leading to avoidance behavior [17–19]. Patients with DFA often report more intense pain compared to less anxious individuals, regardless of the actual invasiveness of the procedure [20]. These findings emphasize the importance of comprehending how early dental experiences, procedural trauma, and cognitive-emotional responses shape DFA across the lifespan.

In contrast, positive dental experiences and regular, non-traumatic visits act as protective factors against DFA, consistent with the latent inhibition hypothesis [21, 22]. Repeated positive exposure builds familiarity and reduces the likelihood of developing fear, whereas lacking such experiences increases vulnerability when painful procedures occur [9]. Current DFA-reduction strategies highlight the effectiveness of cognitive-behavioral methods, including cognitive restructuring, exposure techniques, and relaxation [23, 24]. Beyond past experiences, positive role models, oral-hygiene habits, and strong dental self-efficacy also influence attitudes toward dental care and help lower DFA [25].

Although injection fear, gender, and past dental trauma are known predictors of DFA, few studies have examined their predictive value across different timeframes within an integrated model. By considering childhood, cumulative, and recent dental experiences, this study explores whether recent encounters have a stronger impact on DFA and can outweigh earlier negative events. This temporal perspective offers insight into the dynamic nature of DFA and may help refine targeted interventions and improve prevention and treatment. For example, clinicians may prioritise enhanced support, pain control, and follow-up immediately after encounters to reduce DFA or prevent the development of it [15, 23–25]. Cognitive-behavioral methods, along with educational efforts that foster positive dental experiences, can help reduce DFA, while identifying specific risk factors supports more personalized prevention and encourages regular dental attendance. Thus, systematically identifying patients whose anxiety emerges or worsens after a recent adverse visit can help target these evidence-based strategies and preventive efforts to replace distressing experiences with consistent positive ones [26–28].

Although previous studies have identified early and cumulative traumatic dental experiences as significant contributors to DFA, the relative strength of early versus proximal experiences was not directly compared within a multivariate framework. Existing research focuses on childhood or adult experiences in isolation, or on specific triggers such as injections or extractions, without evaluating how these experiences compete or interact across temporal scales. Accordingly, it remains unclear whether recent dental encounters merely add to prior vulnerabilities or can override them, thereby reshaping DFA in adulthood. Considering these theoretical and empirical foundations, the present cross-sectional study aimed to investigate the associations between DFA and previous dental experiences in three different time dimensions, alongside with fear of pain, and sociodemographic characteristics in an adult population. Specifically, we sought to identify the relative contributions of recent and past direct traumatic experiences, pain-related self-perceptions with sex and age as sociodemographic factors to DFA severity. To provide a more comprehensive analysis, dental experiences were categorized into three time-based dimensions: early (childhood or first encounters), cumulative past, and the most recent dental visit. With this temporal dimension, we intended to explore the relative impact of early or repeated negative experiences, and the most recent dental encounter on DFA severity. To our knowledge, no previous study has evaluated childhood, cumulative past, and most recent dental experiences simultaneously while also adjusting for demographic factors and pain-related fear using three validated DFA measures. By integrating multiple timeframes and multiple instruments, this study aims to test whether DFA can be characterised like a stable, trait-like construct rooted in early experiences, or a state-like construct shaped primarily by recent encounters.

Based on the existing literature, following predictions were formulated:

1. Higher levels of DFA will be associated with more frequent reports of previous traumatic or painful dental experiences.
2. Women and individuals with lower frequency of regular dental visits will report higher DFA compared to men and those attending regular check-ups.
3. Fear of injection-related pain will be the primary predictor of DFA.

By examining these hypothesized associations, we aim to refine the understanding of the multifactorial character of DFA and provide insights for designing more targeted prevention and intervention strategies to reduce DFA.

## Methods

### Participants

The minimum required sample size required was determined by calculating the estimated statistical power for linear regression with a small effect size and a conservative approach (f^2^=0.02, β > 0.95, alpha = 0.05) using the Free Statistics Calculators v4.0 online software available at https://www.danielsoper.com/statcalc/calculator.aspx?id=16. The analysis indicated a minimum required sample size of 769. The full survey can be accessed at https://osf.io/n2hu5/files/gkz53.

All participants were recruited via the Internet by posting invitations on various forums and mailing lists in order to obtain a non-clinical, heterogeneous sample. Only fully completed surveys were stored and analyzed, thus uncomplete responses were excluded from the analyses. None of the subjects reported having been diagnosed with a specific phobia by a physician or psychiatrist. Subjects participated voluntarily and answered the survey online. The research was approved by the Hungarian United Ethical Review Committee for Research in Psychology and was conducted in accordance with the World Medical Association’s Code of Ethics (Declaration of Helsinki). Written informed consent was obtained from all participants.

### Questionnaires

#### Sociodemographic questions and prior experience

Sociodemographic data included age and sex, and questions about prior personal experiences such as whether participants had or had not severe dental problems (requiring multiple treatments) until the age of 12 (childhood). Participants were also asked to select from a list the most invasive procedure they have received in the past 12 months (recent) and ever since being an adult (adulthood). We also asked participants to rate how painful or distressing their last visit to the dentist was on a five-point scale (from 0 - not at all to 5 - extremely).

#### Measurements

Dental anxiety (and fears) were measured with three separate questionnaires. The use of three separate questionnaires to measure dental anxiety strengthens the study by providing a more comprehensive assessment of the construct and increases the reliability of the results through triangulation. The questionnaires we used were the Dental Anxiety Question (DAQ), the Short Dental Fear Question (SDFQ), and the Modified Dental Anxiety Scale (MDAS).

#### Dental Anxiety Question (DAQ)

For this question, participants rate on a three-point scale [29]. It focuses on general, global dental anxiety with asking whether a person is afraid of going to the dentist overall, providing a broad snapshot of their overall fear level without specifying situations or triggers. The original study of the DAQ reported 93% agreement between the single-item question and the Modified Dental Anxiety Scale (see details below); the kappa coefficient was 0.63, the specificity was 0.95, and the sensitivity was 0.80, suggesting that it has good validity and is a psychometrically sound measure.

#### Short Dental Fear Question (SDFQ)

SDFQ is rated on a four-point scale and refers to fear related to the most recent dental visit [30]. Its emphasis is on recency and how the last dental experience influenced the participant’s current level of fear, making it more sensitive to recent negative or positive encounters. The original study of the SDFQ reported Spearman correlations between the SDFQ and other, longer measures of dental anxiety ranging from 0.69 to 0.79, suggesting that the SDFQ captures the essence of these other instruments and may be suitable for measuring dental anxiety. Both the DAQ and the SDFQ are single-item questionnaires.

#### Modified Dental Anxiety Scale (MDAS)

MDAS is a five-item questionnaire that evaluates the emotional reactions of the respondents to different situations (e.g., waiting, scaling) during a dental visit, rated on a five-point scale [31]. It measures situational dental anxiety across different steps of the dental visit, providing a more detailed, multidimensional understanding of the triggers of DFA. In the original study of the MDAS, the Kaiser-Meyer-Olkin value was 0.842, indicating the presence of sufficient common variance to merit factor analysis, and the eigenvalue of 3.69 showed a clear unidimensional factor structure and that the scale could be considered unidimensional. The goodness of fit statistics showed excellent agreement between the model and the raw data (x^2^ = 3.89; CFI=0.999, TLI=0.997, RMSEA=0.031). The internal consistency coefficient of the scale was excellent (0.957, and the 95%CI being 0.953–0.961). The McDonald’s omega for MDAS in the present study was 0.89. For all three measures, higher scores meant greater dental anxiety.

### Statistical analysis

A hierarchical linear regression approach was used to examine the predictive values of various factors measuring prior experience on dental anxiety scores. The analysis was conducted separately for all three DFA questionnaires (DAQ, SDFQ, and MDAS), these always served as the dependent variable in the model. For each dependent variable, we tested three models, depending on the recency of the experience: Model 1 focused on the past year, Model 2 focused on adulthood in general, and Model 3 focused on childhood. Each model consisted of three levels. At Level 1, we entered the variable related to the question about past experience. In Model 1, this was the most invasive procedure they had in the past year, in Model 2, the most invasive procedure they had in adulthood, and in Model 3, whether they had severe dental problems in childhood. At Level 2, we included age and sex as control variables. At Level 3, we entered the self-reported pain and distress experienced during the last dental visit. Tukey-corrected pairwise comparisons were conducted to separate significant main effects for the ordinal variables entered at Level 1. All model assumptions were met, residuals were normally distributed, multicollinearity (VIF values were less than 4) and no issues with heteroscedasticity were detected. All data analyses were performed using JASP statistical software, version 0.19.3.

## Results

We recruited 802 participants (625 women) between the ages of 18 and 72 years (M = 28.74, SD = 9.15). Table 1 shows the detailed descriptive statistics of the questionnaires and more details about the sample.

**Table 1:** Detailed descriptive statistics of the participants and variables included in the study

		Descriptives					Reliability	Correlations					
		Ratio	Mean	SD	Skew-ness	Kur-tosis	McDonald’s ω	1	3	4	5	6	7
1	Age		28.7	9.15	1.16	1.32		—					
2	Sex	77.93% female											
3	MDAS		12.4	4.89	0.463	−0.46	0.887	−0.094**	—				
4	SDFQ		1.77	0.768	1.56	4.89		−0.061	0.654***	—			
5	DAQ		1.68	0.638	0.397	−0.69		−0.024	0.704***	0.510***	—		
6	Most invasive procedure ever		4.73	1.85	−0.793	−0.03		0.302***	0.018	0.100	0.096**	—	
7	Most invasive procedure past year		2.04	2.29	0.719	−0.8		0.038	−0.013	0.098**	0.001	0.327***	—
8	Serious problem in childhood (< 12 y/o)	45.137% yes											
9	Last visit pain and distress		2.42	1.17	0.507	−0.62		0.011	0.503***	0.438***	0.454***	0.171***	0.064

* *p* <.05, ** *p* <.01, *** *p* <.001

### Past year

As an initial step, the effect of dental experiences in the past year on DFA was tested. Detailed statistical results are shown in Table 1, and pairwise comparisons are shown in Appendix 1. Model 1, which included the most invasive procedure in the past year as an independent predictor, was significant for SDFQ and DAQ (but not MDAS), indicating a positive association between the variables. Including age and sex of the participants did not change these predictive patterns (see model 2), but sex emerged as a significant predictor, with women scoring higher than men in both cases. For MDAS, both sex and age (negative association) were significant predictors. In model 3, an additional independent predictor, pain and distress at last visit, was significantly positively associated with the outcome variable in all cases. For MDAS, the significant predictors were sex, age, and last visit. For SDFQ, the significant predictors were the most invasive procedure in the past year, sex, and last visit. Finally, for DAQ, the significant predictors were sex and last visit (Table 2).

**Table 2:** Associations of most invasive procedure in the past year, age, sex, and pain and distress felt during last visit with three DFA questionnaires

Model 1 - Past year	Model statistics						MDAS		
	*R* ^2^	ΔR^2^	RMSE	df	F		b	β	*p*
Level 1	0.005		4.86	7, 794	1.60	Most invasive procedure			0.131
Level 2	*0.034*	0.031***	*4.78*	*9*,* 792*	*4.12*	Most invasive procedure			0.173
						Age	*−0.042*	*−0.078*	*0.028*
						Sex	*1.774*	*0.362*	*< 0.001*
Level 3	*0.277*	0.242***	*4.13*	*10*,* 791*	*31.72*	Most invasive procedure			0.346
						Age	*−0.043*	*−0.080*	*0.009*
						Sex	*1.54*	*0.315*	*< 0.001*
						Last visit pain and distress	*2.081*	*0.498*	*< 0.001*
							SDFQ		
Level 1	*0.026*		*0.754*	*7*,* 794*	*4.01*	Most invasive procedure			*< 0.001*
Level 2	*0.042*	0.018***	*0.747*	*9*,* 792*	*4.85*	Most invasive procedure			*< 0.001*
						Age	−0.0046	−0.055	0.120
						Sex	*0.219*	*0.285*	*< 0.001*
Level 3	*0.199*	0.156***	*0.682*	*10*,* 791*	*20.86*	Most invasive procedure			*0.032*
						Age	−0.0048	−0.057	0.078
						Sex	*0.1895*	*0.247*	*0.001*
						Last visit pain and distress	*0.263*	*0.4007*	*< 0.001*
							DAQ		
Level 1	*0.011*		*0.632*	*7*,* 794*	*2.23*	Most invasive procedure			*0.030*
Level 2	*0.037*	0.028***	*0.622*	*9*,* 792*	*4.39*	Most invasive procedure			*0.038*
						Age	−0.0007	−0.011	0.762
						Sex	*0.258*	*0.404*	*< 0.001*
Level 3	*0.226*	0.189***	*0.558*	*10*,* 791*	*24.98*	Most invasive procedure			0.251
						Age	−0.0009	−0.013	0.683
						Sex	*0.231*	*0.363*	*< 0.001*
						Last visit pain and distress	*0.24*	*0.44*	*< 0.001*

Adjusted R^2^ values are presented. All standardized and unstandardized estimates were calculated from 5000 bootstraps. *** *p* <.001

### Adulthood

**Fig. 1:**
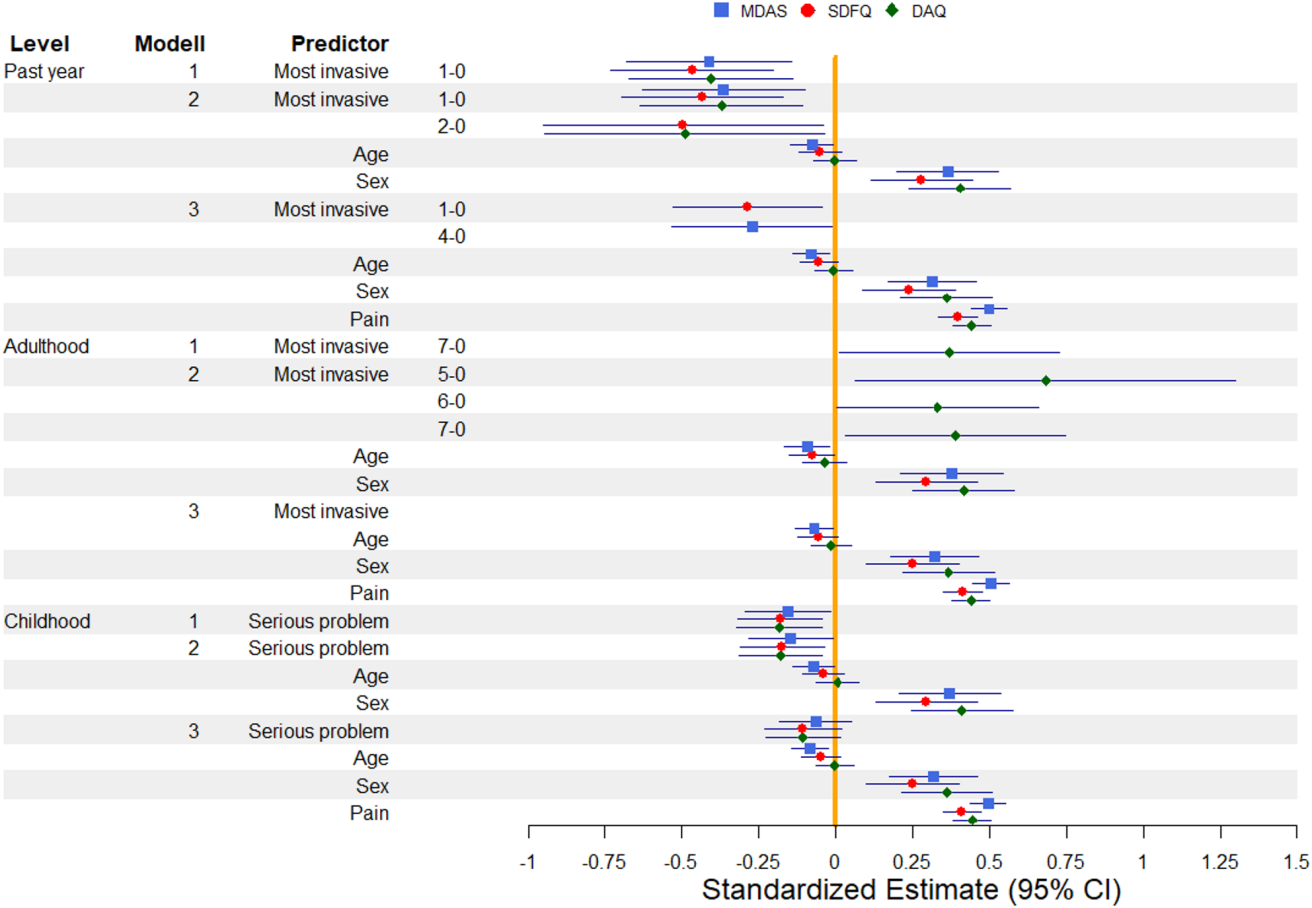
The figure shows the Standardized Estimate (CI95%) values for the three hierarchical linear regressions. On the left side of the figure are the Levels (Past year, Adulthood, Childhood), the Models (1,2, or 3) and the Predictors (Most invasive= most invasive procedure they had, Age, Sex, Pain=Last visit pain and distress, Severe problem=whether they had severe dental problems in childhood). Standard Estimates (CI95%) are shown on the right. The blue square indicates values for MDAS, the red circle for SDFQ, and the green diamond for DAQ. Note: For the variable ‘Most invasive procedure’, which included eight categories and therefore numerous pairwise comparisons, only the statistically significant comparisons are displayed to maintain clarity. The absence of a questionnaire (DAQ, SDFQ or MDAS) from a given model or level indicates that no significant associations were found for that scale in that part of the analysis

After testing recent experiences, the effect of adult dental experience on DFA were analyzed. Detailed statistical results are shown in Table 3, and pairwise comparisons are shown in Appendix 1. Model 1, which included the most invasive procedure during adulthood as an independent predictor, was significant for DAQ (but not for SDFQ and MDAS), indicating a positive association between the variables. This result did not change in Model 2, where age and sex of the participants were included as variables. Sex emerged as a significant predictor, with females scoring higher than males on all three DFA measures and age was negatively associated with MDAS and SDFQ, but not with DAQ. Pain and distress at last visit was also entered as independent predictor (Model 3) and showed significant positive associations with the outcome variable in all cases. For MDAS, the significant predictors were sex, age, and last visit. For SDFQ, the significant predictors were sex and last visit. Finally, for the DAQ, the significant predictors were sex and last visit (Fig. 1).

**Table 3:** Associations of most invasive procedure during adulthood, age, sex, and pain and distress felt during last visit with three DFA questionnaires

Model 2 - Adulthood	Model statistics						MDAS		
	*R* ^2^	ΔR^2^	RMSE	df	F		b	β	*p*
Level 1	−0.003		4.88	7, 794	0.670	Most invasive procedure			0.697
Level 2	*0.0304*	0.0355***	*4.79*	*9*,* 792*	*3.794*	Most invasive procedure			0.384
						Age	*−0.052*	*−0.097*	*0.009*
						Sex	*1.836*	*0.375*	*< 0.001*
Level 3	*0.2745*	0.242***	*4.14*	*10*,* 791*	*31.31*	Most invasive procedure			0.666
						Age	*−0.038*	*−0.072*	*0.026*
						Sex	*1.576*	*0.322*	*< 0.001*
						Last visit pain and distress	*2.099*	*0.502*	*< 0.001*
							SDFQ		
Level 1	0.002		0.763	7, 794	1.22	Most invasive procedure			0.287
Level 2	*0.0235*	0.024***	*0.754*	*9*,* 792*	*3.14*	Most invasive procedure			0.093
						Age	*−0.007*	*−0.083*	*0.026*
						Sex	*0.232*	*0.302*	*< 0.001*
Level 3	*0.18860*	0.1643***	*0.687*	*10*,* 791*	*19.62*	Most invasive procedure			0.610
						Age	−0.005	−0.062	0.068
						Sex	*0.198*	*0.258*	*< 0.001*
						Last visit pain and distress	*0.271*	*0.413*	*< 0.001*
							DAQ		
Level 1	*0.00943*		*0.632*	*7*,* 794*	*2.09*	Most invasive procedure			*0.042*
Level 2	*0.04022*	0.033***	*0.621*	*9*,* 792*	*4.73*	Most invasive procedure			*0.013*
						Age	−0.0031	−0.0442	0.232
						Sex	*0.2657*	*0.4162*	*< 0.001*
Level 3	*0.22408*	0.183***	*0.558*	*10*,* 791*	*24.13*	Most invasive procedure			0.461
						Age	−0.00155	−0.022	0.505
						Sex	*0.2362*	*0.3701*	*< 0.001*
						Last visit pain and distress	*0.23775*	*0.4361*	*< 0.001*

Adjusted R^2^ values are presented. All standardized and unstandardized estimates were calculated from 5000 bootstraps. *** *p* <.001

### Childhood experiences

The effect of childhood dental experiences on DFA was also tested. Detailed statistical results are presented in Table 3, and pairwise comparisons are presented in Appendix 1. Model 1, which included whether the participant had a self-reported severe dental problem in childhood as an independent predictor, was significant for all measures of DFA. Those with severe dental problems in childhood scored higher than those without such history. Two additional variables, age and sex were added to the analysis, but having severe dental history in childhood remained a significant predictor (see Model 2). Sex also as found to be an independent predictor, with females scoring higher than males on all three DFA measures. Age was negatively associated with MDAS, but not with SDFQ or DAQ. Additionally, pain and distress at last visit, was included to the analysis and showed significant positive associations with the outcome variable in all cases. For MDAS, the significant predictors were sex, age, and last visit. For SDFQ, the significant predictors were sex and last visit. Finally, for the DAQ, the significant predictors were sex and last visit (Table 4).

**Table 4:** Associations of severe dental problem in childhood, age, sex, and pain and distress felt during last visit with three DFA questionnaires

Model 1 - Childhood	Model statistics						MDAS		
	*R* ^2^	ΔR^2^	RMSE	df	F		b	β	*p*
Level 1	*0.006*		*4.87*	*1*,* 800*	*5.86*	Serious problem	*−0.838*	*−0.171*	*0.016*
Level 2	*0.035*	0.0315***	*4.8*	*3*,* 798*	*10.73*	Serious problem	*−0.790*	*−0.1615*	*0.021*
						Age	*−0.039*	*−0.0747*	*0.033*
						Sex	*1.804*	*0.3687*	*< 0.001*
Level 3	*0.277*	0.2419***	*4.15*	*4*,* 797*	*77.74*	Serious problem	−0.387	−0.0790	0.193
						Age	*−0.044*	*−0.0814*	*0.007*
						Sex	*1.552*	*0.3171*	*< 0.001*
						Last visit pain and distress	*2.065*	*0.4940*	*< 0.001*
							SDFQ		
Level 1	*0.0077*		*0.764*	*1*,* 800*	*7.17*	Serious problem	*−0.145*	*−0.189*	*0.008*
Level 2	*0.024*	0.019***	*0.756*	*3*,* 798*	*7.57*	Serious problem	*−0.1399*	*−0.182*	*0.010*
						Age	−0.0037	−0.045	0.205
						Sex	*0.2309*	*0.301*	*< 0.001*
Level 3	*0.192*	0.169***	*0.688*	*4*,* 797*	*48.70*	Serious problem	−0.087	−0.113	0.077
						Age	−0.004	−0.050	0.117
						Sex	*0.198*	*0.258*	*< 0.001*
						Last visit pain and distress	*0.271*	*0.413*	*< 0.001*
							DAQ		
Level 1	*0.008*		*0.635*	*1*,* 800*	*7.51*	Serious problem	*−0.124*	*−0.194*	*0.006*
Level 2	*0.035*	0.029***	*0.625*	*3*,* 798*	*10.61*	Serious problem	*−0.120*	*−0.188*	*0.007*
						Age	*−1.98e-4*	*0.003*	*0.935*
						Sex	0.262	0.41	*< 0.001*
Level 3	*0.227*	0.192***	*0.560*	*4*,* 797*	*59.68*	Serious problem	−0.073	−0.114	0.069
						Age	*−6.19e − 4*	*−0.009*	*0.777*
						Sex	0.232	0.364	*< 0.001*
						Last visit pain and distress	*0.240*	*0.440*	*< 0.001*

Adjusted R2 values are presented. All standardized and unstandardized estimates were calculated from 5000 bootstraps. *** *p *<0.001

## Discussion

This study aimed to investigate the impact of three different temporal dimensions of previous dental experiences, along with self-perceptions of pain, and sociodemographic factors on DFA. Our results support the multifactorial nature of DFA and align with previous research emphasizing the contributions of experiential and cognitive-emotional factors to the development of DFA [1, 2, 10]. Notably, the experiences related to the most recent dental visit emerged as the most influential factor in shaping individuals’ current level of DFA. While early and past experiences certainly also impact DFA, our findings suggest that the most recent experience may override these prior influences and primarily affect the actual intensity of DFA. This finding is a key contribution of this study and demonstrates, that recent dental experiences may exert a stronger influence on current DFA than earlier events. This supports the idea that DFA is not only shaped by conditioning and early encounters, but may also be modified by proximal experiences, highlighting the importance of patient-centred approaches in clinical dental care. These main associations also raise important questions about the trait-like versus state-like nature of DFA, since it indicates that dental fear and anxiety may not be a purely stable, enduring characteristic (trait), but rather a dynamic response shaped by recent experiences (state).

This study addresses an underexplored topic in regard of DFA, that is, whether recent dental experiences retain a uniquely strong influence when earlier traumatic events are accounted for simultaneously. Prior work has demonstrated that both early and adult negative experiences contribute to DFA, but these temporal dimensions have rarely been examined within the same predictive model. By showing that recent pain and distress remain the dominant predictor even after adjusting for childhood and cumulative experiences, our findings clarify the temporal dynamics of DFA more precisely than previous research.

Consistent with the first prediction, individuals reporting previous traumatic or painful dental experiences exhibited elevated DFA compared to individuals with no or minor previous stressful experiences. This association, that aversive dental experiences are significant indicators of developing DFA reinforces the classical conditioning hypothesis showing that former negative experiences increase vulnerability to DFA [9, 32]. However, traumatic dental experiences were not the only significant predictor for high DFA, suggesting the relevance of additional cognitive-emotional factors, as shown in previous studies [10, 12].

Aligning with prior studies and our second prediction, women reported higher DFA levels than men, reinforcing the presence of gender differences in anxiety sensitivity and pain perception [4, 5]. Furthermore, individuals with irregular dental visit history also reported higher DFA compared to individuals with consistent dental attendance patterns suggesting that regular dental visits, especially if associated with positive experiences, could serve as a protective factor against DFA [6, 21].

Finally, fear of injection-related pain emerged as a highly significant predictor of DFA, emphasizing the unique contribution of pain-related and needle-related fears to avoidance behaviors [17–19]. The anticipated or actual pain during dental injections appear to intensify DFA, which underlines the importance of pain management strategies and patient communication in reducing dental fear and anxiety.

Our findings align with recent literature emphasizing the impact of proximal experiences over cumulative past events in forming anxiety-related behaviors. For instance, a recent study incorporating machine learning approaches highlighted the dominance of immediate pain and injection-related fears over early-life dental experiences in predicting the level of severity of DFA [15]. Moreover, systematic reviews have showed that traumatic dental experiences in both childhood and adulthood increase the risk of DFA, but more recent distress might modulate or change these early influences [2, 11]. These findings suggest that interventions aimed at reframing recent dental experiences as positive could be effective in reducing DFA [16, 23]. Further studies with more systematic and longitudinal design are needed to unravel the temporal aspects of DFA and validate targeted interventions.

Several strengths of the present study should be highlighted. Our findings rely on validated instruments, and the incorporation of different temporal aspects of experiential, and conditioning factors on a relatively large adult sample improve the generalizability of these findings. Nevertheless, this study is not without limitations. The cross-sectional design and self-reported measures limit causality along with establishing temporal changes and are prone to recall bias or social desirability. Future studies with longitudinal design should be conducted to explore and refine causal pathways and long-term effects of tailored interventions on DFA. The online recruitment method may also have led to a bias in selection of participants, as individuals with severe DFA might have been less likely to participate due to their heightened avoidance of dental-related topics. This potential underrepresentation of highly anxious individuals could potentially influence the strength of the observed associations. Future studies should include other recruitment methods, such as clinical sampling to better capture this subgroup. Another important aspect to include in future studies is to register experiences in adolescence. The current study did not ask about dental problems or procedures experienced between 13 years and becoming an adult (18-year-old). Furthermore, since the study relied on retrospective self-reports, participants’ current dental fear may have influenced how they remembered earlier experiences, potentially introducing mood-congruent recall bias. Moreover, despite controlling for the effects of participants’ age, the recall of previous, but not recent experiences may also be affected by the time passed since it happened. Therefore, the associations between prior events and DFA should be interpreted with caution, and longitudinal studies are needed to confirm the temporal direction of these relationships. An additional limitation is the gender-related imbalance of the sample. Although we included gender as a variable in our analyses to account for potential bias, the unequal gender distribution in our sample may still limit the extent to which our findings can be generalised. The same concerns may arise in relation to different age groups, geographical areas and populations. Furthermore, this study did not assess systemic medical or psychiatric comorbidities or other potential confounding factors, which may influence pain perception and dental anxiety. Future studies should include such factors to define the specific effects of dental experiences compared to broader health-related variables.

## Conclusions

This study highlights the importance of traumatic experiences, conditioning processes, and sociodemographic factors in relation to DFA, underlining its multifactorial nature. To reshape individual’s cognitive appraisals linked to negative experiences, or to manage pain effectively, and to promote positive dental experiences should be targeted by interventions to effectively reduce dental fear and anxiety and related oral health consequences. Furthermore, future research should focus on whether most recent positive dental experiences are indeed dominating over prior negative ones and sufficient to reduce associated fear and anxiety. Besides gaining deeper insights into the dynamic nature of DFA, such findings would be also helpful in developing and refining therapeutic strategies to disrupt maladaptive fear cycles. Improving dental health literacy and reducing fears related to dental care seem to be influential public health strategies to decrease dental fear and anxiety.

## Data Availability

The dataset supporting the conclusions of this article are available in the Open Science Framework repository, [https://osf.io/n2hu5/]. Other data from this sample have been reported in a previous publication, but the current manuscript includes new analyses with factors and data not published previously.

## Abbreviations

CFI: Comparative fit index
DAQ: Dental Anxiety Question
DFA: Dental fear and anxiety
MDAS: Modified Dental Anxiety Scale
RMSE: Root mean square error
RMSEA: Root Mean Square Error of Approximation
SDFQ: Short Dental Fear Question
TLI: Tucker-Lewis Index
VIF: Variance Inflation Factor
